# Identifying Critical State of Complex Diseases by Single-Sample-Based Hidden Markov Model

**DOI:** 10.3389/fgene.2019.00285

**Published:** 2019-04-04

**Authors:** Rui Liu, Jiayuan Zhong, Xiangtian Yu, Yongjun Li, Pei Chen

**Affiliations:** ^1^School of Mathematics, South China University of Technology, Guangzhou, China; ^2^Shanghai Jiao Tong University Affiliated Sixth People's Hospital, Shanghai, China; ^3^School of Computer Science and Engineering, South China University of Technology, Guangzhou, China

**Keywords:** hidden Markov process, single-sample-based diagnosis, dynamical network biomarker (DNB), pre-disease state, critical transition, early-warning signal

## Abstract

The progression of complex diseases is generally divided as a normal state, a pre-disease state or tipping point, and a disease state. Developing individual-specific method that can identify the pre-disease state just before a catastrophic deterioration, is critical for patients with complex diseases. However, with only a case sample, it is challenging to detect a pre-disease state which has little significant differences comparing with a normal state in terms of phenotypes and gene expressions. In this study, by regarding the tipping point as the end point of a stationary Markov process, we proposed a single-sample-based hidden Markov model (HMM) approach to explore the dynamical differences between a normal and a pre-disease states, and thus can signal the upcoming critical transition immediately after a pre-disease state. Using this method, we identified the pre-disease state or tipping point in a numerical simulation and two real datasets including stomach adenocarcinoma and influenza infection, which demonstrate the effectiveness of the method.

## Introduction

**Figure 1:**
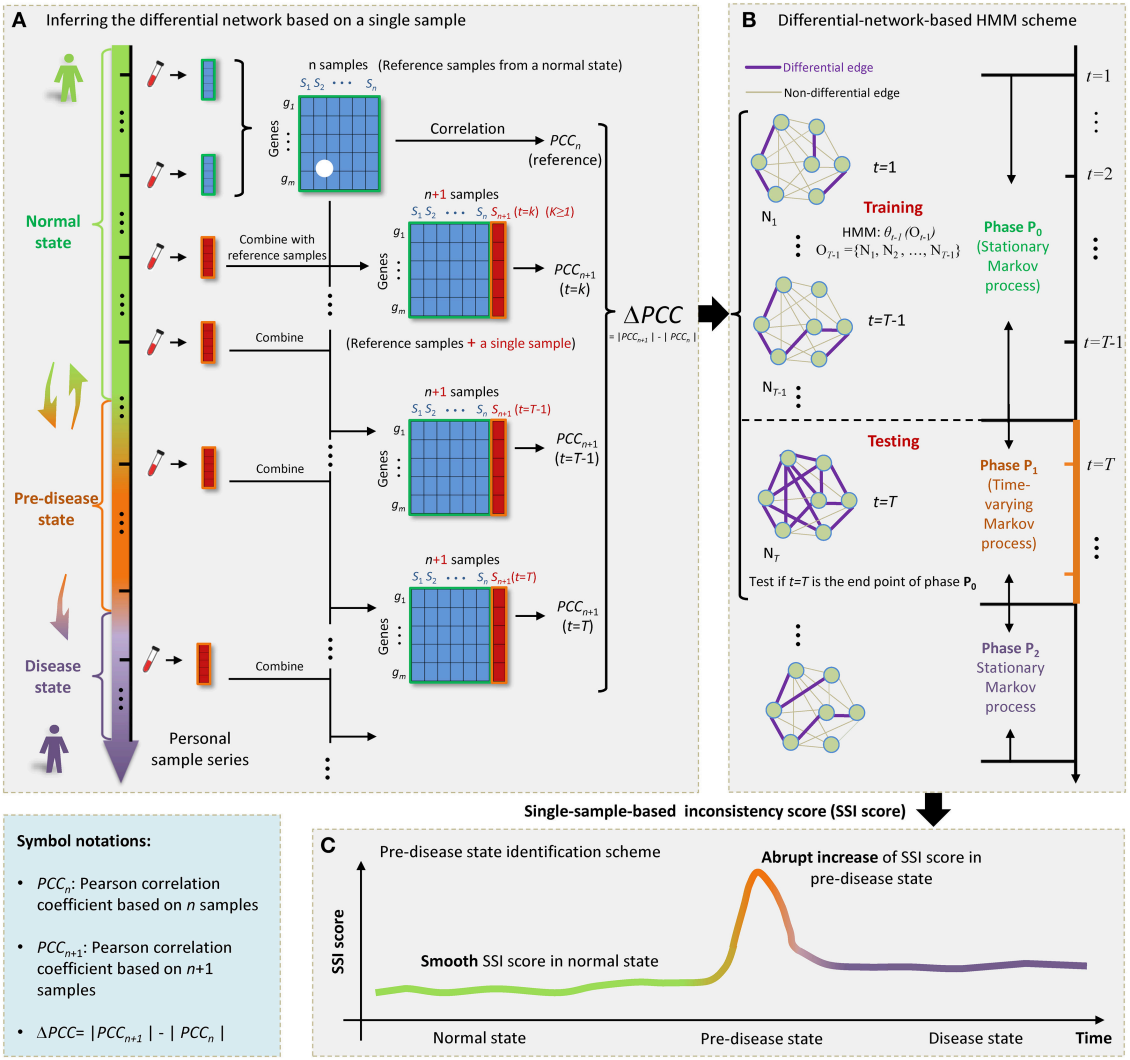
The outline for identifying the SSI score based on HMM. **(A)** The progression of complex diseases is generally modeled as three states, i.e., a normal state, a pre-disease state, and a disease state. The pre-disease state is immediately before the sudden deterioration, which is sensitive to treatment and reversible to the normal state. The disease state is usually irreversible even with intensive medical care. For an individual, samples from a few initial time points can be regarded as reference. Each single case sample was added to the reference, forming a series of combining samples. **(B)** At each time point *t* = 1, 2, … *T*, a differential network N_*t*_ was constructed by PCC. **(C)** The sharp increase of SSI score signals the upcoming critical transition into the disease state.

Considerable evidence suggests that during the progression of many complex diseases the deterioration is not necessarily smooth but abrupt (Litt et al., 2001; McSharry et al., 2003; Scheffer et al., 2009). In order to describe the underlying mechanism of complex diseases, their evolutions are often modeled as time-dependent non-linear systems, in which the abrupt deterioration is viewed as the phase transition at a tipping point (Murray, 2002; Venegas et al., 2005; Hirata et al., 2010; He et al., 2012; Liu et al., 2012). Therefore, from a dynamical systems' perspective, the general progression of complex diseases was modeled as three states or stages (Figure 1A): (i) a normal state, which represents a relative healthy stage with high stability and robustness to perturbations; (ii) a pre-disease state, which was defined as the limit of the normal state, and locating just before the occurrence of sudden deterioration, therefore, with low stability and robustness; (iii) a disease state, which represents a serious deteriorated stage generally with high stability and robustness, because it is usually very difficult to return to the normal state even with intensive treatment (Liu et al., 2014a). In contrast to the irreversible disease state, the pre-disease state is sensitive to perturbation and thus reversible to the normal state if timely and appropriate treatment is received during this stage. It is thus crucial to detect the pre-disease state for patients with complex diseases. However, it is hard to detect a pre-disease state by traditional biomarkers since it is similar to the normal state in terms of the phenotype and gene expression.

Recently, the dynamical network biomarker (DNB) method was proposed to detect the pre-disease state (Chen et al., 2012), that is, by identifying a group of DNB biomolecules (e.g., genes and proteins) which together signal the occurrence of pre-disease state in the following three ways: (i) the DNB members turn to be widely fluctuating; (ii) the correlation between any two DNB members increase significantly; (iii) the correlation between a DNB member and a non-DNB molecule decrease significantly. Different from traditional biomarkers, DNB aims at signaling the pre-disease state before the occurrence of catastrophic deterioration. This method has been employed by many groups and applied to a number of cases, including detecting the tipping points of cell fate decision (Mojtahedi et al., 2016) and cellular differentiation (Richard et al., 2016) studying immune checkpoint blockade (Lesterhuis et al., 2017) and identifying the critical transition states during various biological processes (Liu et al., 2014b, 2018; Chen et al., 2015, 2017, 2018). However, it is noted that the DNB method works only when there are multiple case samples, so that the above three statistical conditions can be evaluated. This limits the practical application of DNB in many clinical cases because generally it is impossible to collect multiple samples for each individual at a time point.

In this work, by exploring the differential information between the normal and pre-disease states, we proposed a single-sample-based hidden Markov model (HMM) to signal the tipping point, even if there was only one case sample available. Specifically, the normal state was modeled as a stationary Markov process due to its highly stable nature in dynamics, while the pre-disease state was viewed as a time-varying Markov process considering its dynamical instability. Taking multiple normal samples as the references or background, a differential network whose edges carried the differential information before and after combing a single sample with references, was obtained specific to the single sample derived at a time point (Figure 1A). Then, under the hypothesis that a time point *t* = *T* (*T* > 2) is the candidate tipping point, a probabilistic score, namely single-sample-based inconsistency score (SSI score), was developed for quantitatively measuring the difference between samples from a normal state and that from a pre-disease state. The calculation of SSI score was based on an HMM, where the HMM was trained by taking a series of differential networks derived up to *t* = *T*−1 as the training set (Figure 1B). The abrupt increase of such probabilistic score indicates the occurrence of tipping point (Figure 1C). Clearly, this approach is individual-specific, and thus may help to achieve personalized diagnosis based on the historical information of patients. To validate the effectiveness, this method has been applied to a numerical simulation and two real datasets, i.e., stomach adenocarcinoma (STAD) dataset from TCGA database and influenza infection dataset from GEO database.

## Methods

### Theoretical Basis

The theoretical basis of this study is the DNB theory, which provide the following generic properties when a dynamical system approaches a bifurcation point (Chen et al., 2012):

1. SD(*x*) increases sharply, where *x* represents the expression of a DNB member, SD represents the standard deviation.
2. PCC(*x*_1_, *x*_2_) increases sharply, where *x*_1_ and *x*_2_ represent the expressions of any two DNB members, PCC means the Pearson correlation coefficient.
3. PCC(*x, y*) decreases sharply, where *x* and *y*, respectively, represent the expressions of a DNB member and a non-DNB gene.
4. Neither SD(*y*) nor PCC(*y*_1_, *y*_2_) has significant change, where *y, y*_1_ and *y*_2_ represent expressions of non-DNB genes.

The detailed description and derivation of DNB can be seen in reference (Liu et al., 2015) and its Supplementary Information. In view of the dynamical characteristics of the normal state, i.e., stable dynamics with little fluctuation and high resilience, it was modeled as a stationary Markov process. The pre-disease was modeled as a time-varying Markov process due to its highly unstable dynamics with strong fluctuation and low resilience. The disease state can be regarded as another stationary Markov process because of its dynamical stability (Chen et al., 2016). To identify the pre-disease state, it is equivalent to detect a switching point at which a stationary Markov process ends and turns into a time-varying Markov process.

### Algorithm

**Figure 2:**
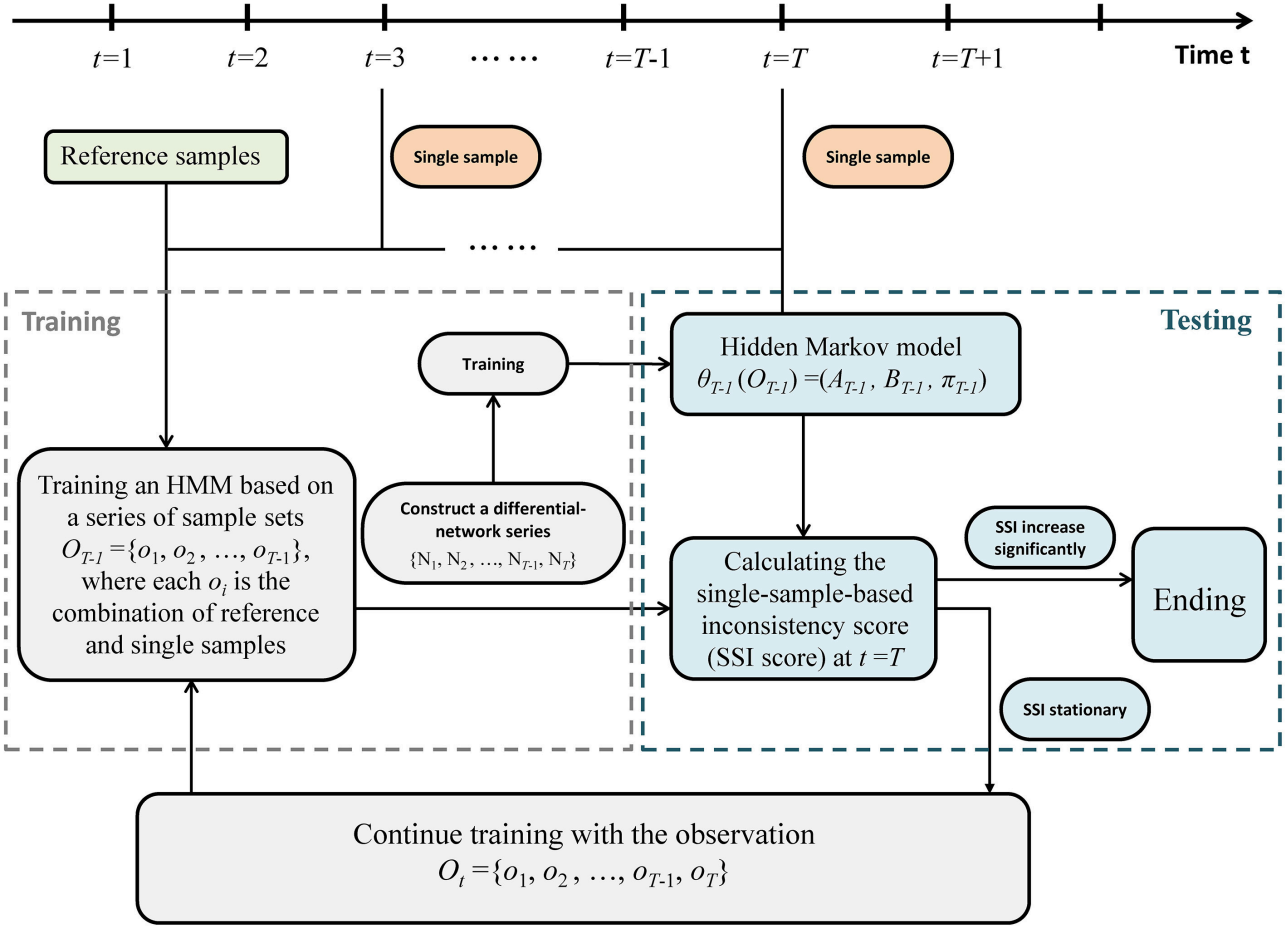
The algorithm of the single-sample-based HMM. The above flowchart shows how the algorithm works based on a series of single case samples. Regarding a point *t* = *T* (*T* > 2) as a candidate tipping point, the sample series is divided into training part ranging from *t* = 1 to *t* = *T*−1, and testing part starting from *t* = *T*. If a probabilistic score (single-sample-based inconsistency score, SSI score) increases significantly, then the candidate *t* = *T* is determined as the identified tipping point, and the algorithm ends. Otherwise, if there is no significant change in SSI score, then *t* = *T* is classified as a time point belonging to the normal state, and the algorithm continues with *t* = *T*+1 being a new candidate tipping point.

A sketch of the single-sample-based HMM algorithm was provided in Figure 2. Specifically, detecting the outset of a pre-disease state is equivalent to identifying the end of this stationary Markov process, which requires a detailed model to present such stationary Markov process. Therefore, an HMM was trained and employed to describe the dynamical characteristics of the system in the normal state. And a probability index was proposed to evaluate the inconsistency between a sample from a testing point and the trained HMM. We carry out the following algorithm to identify the tipping point by using only one case sample.

- **Choosing Reference Samples**
  A few samples that represents the relatively healthy condition were chosen as the reference or background. Generally, for individual-specific samples (e.g., samples for each symptomatic subject in influenza infection dataset), samples from a few initial time points of an individual (as shown in Figure 1A) can be regarded as reference. For stage-course data (e.g., TCGA data for stomach adenocarcinoma), samples from a normal cohort or normal tissue can be viewed as reference.
- **Training Process**
  First, we added each single case sample to the reference (Figure 1A), forming a series of combining samples. In other words, if there were *n* samples in the reference, in each time point we obtained a set of *n* + 1 samples, which can be viewed as a perturbation to *n* samples in the reference group.
  Second, based on the observation samples at each time point *t*, a differential network N_*t*_ was constructed by the difference of the corresponding Pearson correlation coefficient (PCC) between the reference and combined samples (Figures 1A,B), that is,
  $$\begin{array}{l} \Delta \text{PCC}(g_{i},g_{j})=\left|{\text{PCC}}_{n+1}(g_{i},g_{j})\right|\;-\;\left|{\text{PCC}}_{n}(g_{i},g_{j})\right|, \end{array}$$
  Where *g*_*i*_ and *g*_*j*_ represent gene expressions for any pair of genes. Then |Δ*PCC*(*g*_*i*_, *g*_*j*_)| was employed to constructed the differential network, i.e., when |Δ*PCC*(*g*_*i*_, *g*_*j*_)| > *d*, there was a differential link between *g*_*i*_ and *g*_*j*_ (Figure 1B), where threshold *d* was selected based on specific real data, that is, *d* was chosen such that few differential links arising in the initial differential networks of the normal state, thus highlighting the pre-disease state when many links appear. After this step, we obtained a differential-network series {N_1_, N_2_, …, N_*T*_, …}.
  Third, suppose a time point *t* = *T* (*T* > 2) as a candidate tipping point. Then differential-network series was divided into training part ranging from *t* = 1 to *t* = *T*−1, i.e., observation sequence *O*_*T*−1_ = {*o*_1_, *o*_2_, …, *o*_*T*__−1_} = {N_1_, N_2_, …, N_T−1_}, and testing part starting from *t* = *T*, i.e., *o*_*T*_ = {N_*T*_}. Let {*s*_1_, *s*_2_, …, *s*_*t*_} represents the state sequence up to *t*. Symbols *P*_0_ and *P*_1_, respectively, denote the normal state (*P*_0_) and a possible pre-disease state (*P*_1_), which are two unobserved (hidden) states. Then based on the training samples *O*_*T*−1_ = {N_1_, N_2_, …, N_T−1_}, a HMM
  $$\begin{array}{l} \theta^{T-1}\left(O_{T-1}\right)=\left(A^{T-1},B^{T-1},\pi \right) \end{array}$$
  was trained by the Baum-Welch procedures (Bilmes, 1998). Here, the subscript *T*-1 of θ denotes that the HMM θ was obtained from the training samples up to *t* = *T*−1. The state transition matrix at time point *T*−1 is
  $$\begin{array}{l} A^{T-1}={\left(a_{ij}\right)}_{2\times 2} \end{array}$$
  with
  $$\begin{array}{l} a_{ij}=P\left(s_{q}=P_{i}|s_{q-1}=P_{j}\right),i,j\in \left\{\;0,1\right\}. \end{array}$$
  *q* − 1 ∈ {1, …, *T* − 2} stands for a time point in the training process, *q* stands for the next time point after *q* − 1. The observation matrix at time point *T*−1 is
  $$\begin{array}{l} B^{T-1}={\left(b_{jk}\right)}_{2\times N} \end{array}$$
  with
  $$\begin{array}{l} b_{jk}=P\left(\#1\left(q\right)=k|s_{q}=P_{j}\right),j\in \left\{0,1\right\},\;k\in \left\{0,1,\ldots ,M\;\right\}, \end{array}$$
  Where #1(*q*) = *k* represents that there are *k* edges in the differential network N_T−1_, *M* is the number of all possible edges, e.g., $M=C_{m}^{2}$ if there are *m* nodes in N_*q*_. The initial probabilities are
  $$\begin{array}{l} \pi =\left\{\pi_{1},\;\pi_{2}\right\} \end{array}$$
  with π_*i*_ = *P*(*s*_*q* − 1_ = *P*_*i*_), *i* ∈ { 0, 1}.
- **Testing Process**
  Based on the testing sample *o*_*T*−1_ = {N_*T*_} we tested if the candidate point *t* = *T* is a “real” tipping point. A single-sample-based inconsistency score (SSI score) was proposed, i.e.,
  $$\begin{array}{l} SSI\left(T\right)=P\left(s_{T}=P_{1}|s_{1}=P_{0},s_{2}=P_{0},\ldots ,\;s_{T-1}=P_{0};\theta^{T-1}\right)\\ \;=1-P\left(s_{T}=P_{0}|s_{1}=P_{0},s_{2}=P_{0},\ldots ,\;s_{T-1}=P_{0};\theta^{T-1}\right)\\ \;=1-P\left(s_{T}=P_{0}|\;s_{T-1}=P_{0};\theta^{T-1}\right)\\ \;=1-\frac{P\left(s_{T}=P_{0},s_{T-1}=P_{0};\theta^{T-1}\right)}{P\left(s_{T-1}=P_{0};\theta^{T-1}\right)}. \end{array}$$
  Given the HMM θ^*T*−1^, the SSI score was calculated by a forward algorithm. According to above settings, the calculation of probability *SSI*(*T*) (the inconsistency probability) at a time point *t* = *T* only relies on the samples from *T*−1 and *T*. If *SSI*(*T*) increases significantly, then the candidate point *t* = *T* is determined as the identified tipping point, and the algorithm ends (Figure 2). Otherwise, if there is no significant change in *SSI*(*T*), then *t* = *T* is classified as a time point belonging to the normal state. Accordingly, the differential network *o*_*T*_ = {N_*T*_} is added to the training set, and the algorithm continues with *t* = *T*+1 being a new candidate tipping point (Figure 2).

According to the DNB theory, there are few differential edges in a differential network constructed in a normal stage, due to the high stability nature of the system during the normal stage. However, when the system approaches the critical transition point, there are many differential edges appearing in the differential network due to the time-varying and fluctuating dynamics of the system. Specifically, the algorithm is guaranteed by the generic properties 2 and 3 listed in section Theoretical Basis.

### Data Accessing and Processing for Real Datasets

Two gene expression profiling datasets including the time-course dataset for influenza virus infection process (GSE30550) downloaded from the NCBI GEO database (www.ncbi.nlm.nih.gov/geo) and stage-course dataset for stomach adenocarcinoma (STAD) from TCGA database (http://cancergenome.nih.gov). For omics data (GSE30550), we discarded the probes without corresponding NCBI Entrez gene symbol. After removing any redundancy in dataset GSE30550, we obtained 11,451 molecules through probe mapping. For each gene mapped by multiple probes, the average value was employed as the gene expression.

When applied the algorithm to both two disease datasets, there were two extra steps as follows.

First, the expression profiling information was mapped to the protein-protein interaction networks from STRING (http://stringw-db.org) (Szklarczyk et al., 2014) for *Homo sapiens*. In such a network, the edges were filtered by the confidence level with a threshold of 0.700. All the isolated nodes were discarded. Then we choose the cutoff parameter *d* so that there are only 10% edges in the first differential network comparing with original STRING network, that is, over 90% edges disappear comparing with the original STRING network due to the generic property that the network structure would remain stable during the normal stage, and thus there are few edges in a differential network based on samples generated from normal stage.

Second, the differential network was partitioned into local networks to reduce computational complexity. Each local network contained a center node and its first-order neighbors. The local SSI score for each local network was calculated through above algorithm. Given *k* local networks, then a weighted average SSI score was derived as follows,

$$\begin{array}{l} \text{SSI}=\frac{n_{1}{SSI}_{1}+n_{2}SSI_{2}+\ldots +n_{k}SSI_{k}}{n_{1}+n_{2}+\ldots +n_{k}}, \end{array}$$

Where *n*_*i*_ denotes the number of nodes in the *i*-th local network (*I* = 1, 2,…, *k*) and *SSI*_*i*_ stands for the local *SSI* score of this subnetwork.

The networks were visualized using Cytoscape (www.cytoscape.org) and the functional analysis was based on Ingenuity Pathway Analysis (IPA, http://www.ingenuity.com/products/ipa) and KEGG enrichment analysis (http://www.genome.jp/kegg/tool/map/_pathway2.html).

## Results

### Identifying the Critical Transition for a Numerical Simulation Model

**Figure 3:**
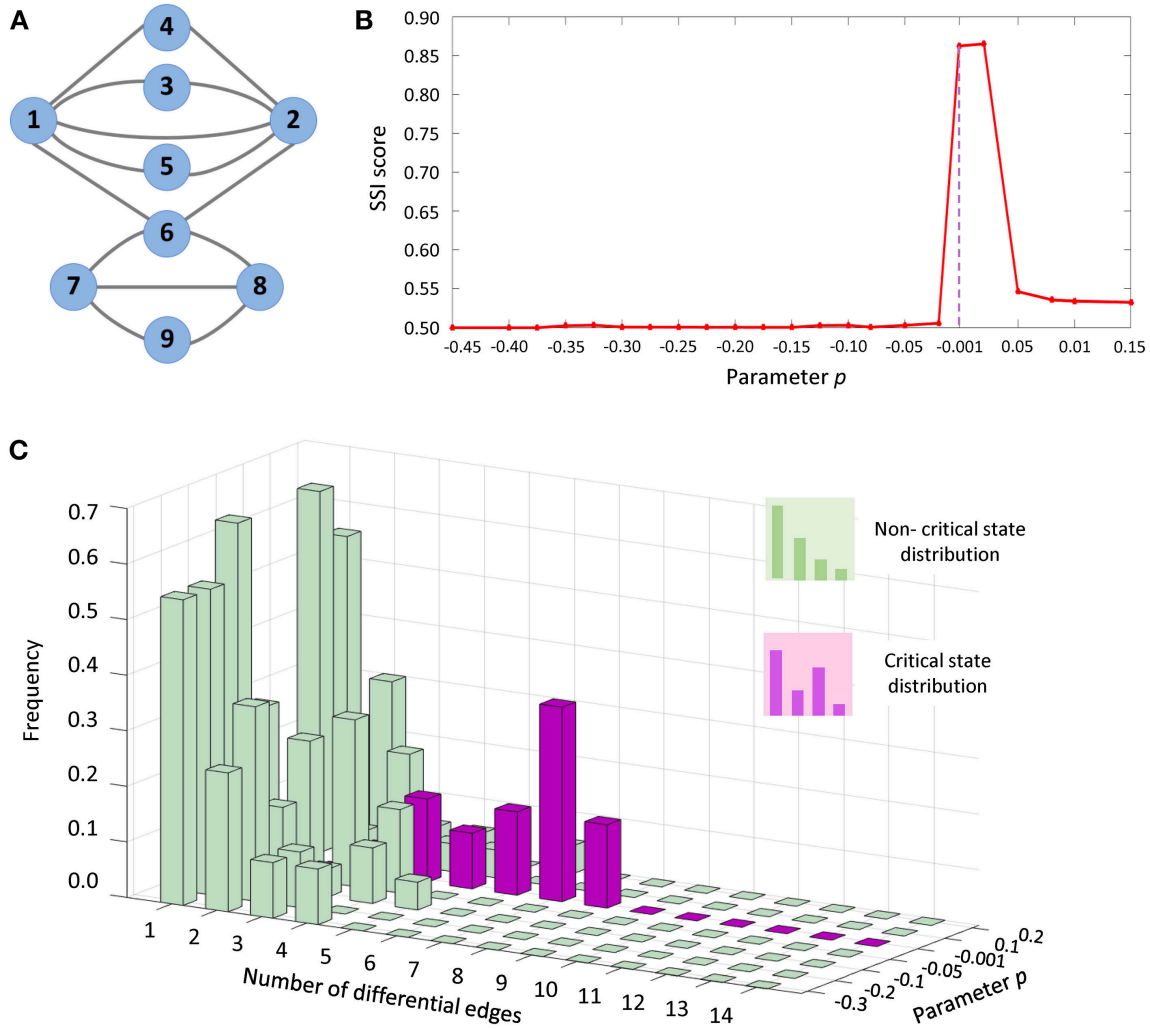
The application of SSI score in numerical simulation. **(A)** The numerical simulation was based on a nine-node regulatory network. **(B)** The abrupt increase of SSI score indicates the tipping point at *P* = 0. **(C)** From the dynamical changes of differential-edge distribution, it is seen that there is a significantly different distribution (purple bars) comparing with others (green bars) when the system approaches the tipping point (*p* = 0).

The proposed computational method and SSI score was applied to a numerical simulation dataset, which was generated from a nine-node regulatory network (Figure 3A) with a set of nine stochastic differential equations Equation (S1) provided in Supplementary Information. Such model of regulatory network of Michaelis-Menten form, is usually employed to study genetic regulations including transcription, translation, diffusion, and translocation processes (Chen et al., 2009). With varying parameter *p* ranging from −0.45 to 0.15, a dataset was generated for numerical simulation.

In Equation (S1), the parameter value *p* = 0 was set as a bifurcation value, at which the system undergoes a critical transition. The dynamical change in SSI score was exhibited in Figure 3B. Clearly, there is an abrupt increase of SSI score when the system approaches the tipping point (*p* = 0). Thus, the significant increase of SSI score indicates the upcoming critical transition at *p* = 0. In Figure 3C, after 1,000 simulations, the distribution of differential edges was illustrated for the network specific to each parameter value. It is seen that the frequency for the occurrence of differential edges was significantly different in the vicinity of the tipping point (*p* = 0), which implies that much more edges would occur in the differential network when the system approaches the tipping point.

### Identifying the Critical Transition for Stomach Adenocarcinoma

Cancer of the stomach is difficult to cure unless it is found at an early stage (before its metastasis). Unfortunately, because early stomach cancer causes few symptoms, the disease is usually advanced when the diagnosis is made (Wadhwa et al., 2013). According to a clinical-stage division (Guide, 2009) stage IV is generally regarded as a severe deteriorated stage, at which cancer has spread to nearby tissues and distant lymph nodes or has metastasized to other organs. Generally, a cure is very rarely possible at stage IV. Therefore, it is important to detect the early-warning signal for metastasis before stage IV.

The proposed method was employed in STAD dataset from TCGA, and identified the tipping point of distant metastasis (IIIA stage). This dataset contained RNA-Seq data and included 141 tumor samples and 33 tumor-adjacent samples. The tumor samples were grouped into seven stages, that is, stage IA (9 samples), stage IB (18 samples), stage IIA (23 samples), stage IIB (29 samples), stage IIIA (27 samples), stage IIIB (20 samples), and stage IV (15 samples) of stomach cancer. The tumor-adjacent samples were regarded as control data and were employed as reference samples.

**Figure 4:**
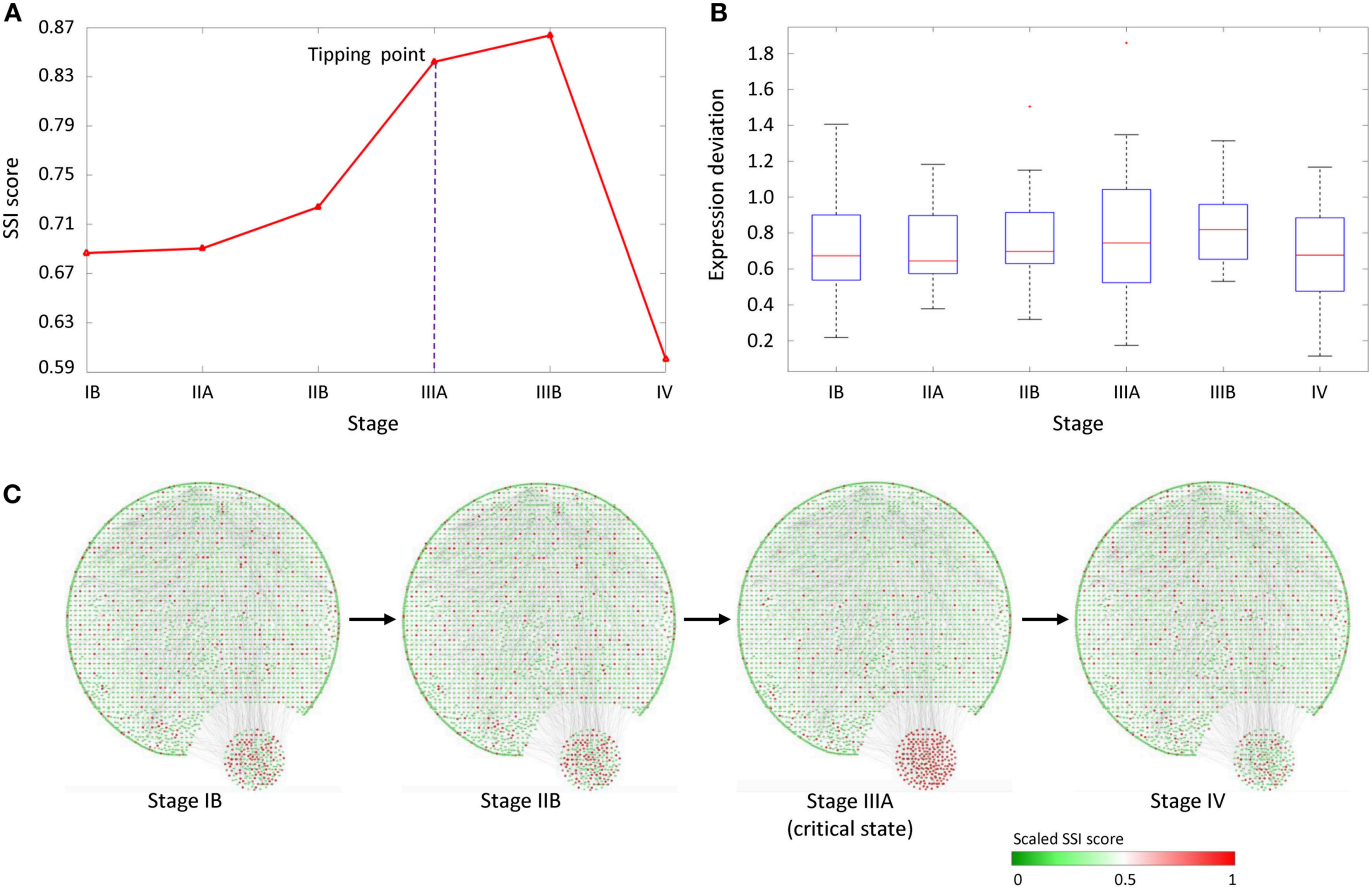
The application of SSI score in STAD dataset. **(A)** The significant increase of SSI score indicates the tipping point at stage IIIA, before the deterioration into distant metastasis at stage IV. **(B)** The average expression deviation between each single sample and the reference. **(C)** The dynamical evolution of the whole gene regulatory network. The top 1% genes with the largest local SSI scores were arranged at the right-bottom corner.

As shown in Figure 4A, the abrupt increase of average SSI score indicated the imminent critical transition in tipping point stage (IIIA), after which cancer would spread to the serosal layer of the stomach wall (stage IIIA) and ultimately cause distant metastasis (stage IV). In Figure 4B, the box plot showed that the expression deviation of deferential expression genes fails to provide any effective signals for the tipping point, where the differential-expression genes were obtained by comparing with tumor-adjacent TA samples at each stage. Figure 4C shows the dynamical evolution of the whole gene regulatory network including 3,247 nodes and 22,301 edges. These edges were selected through the STRING network with high confidence level (level higher than 0.700). A group of 214 nodes, i.e., genes with the most significant increases in their local SSI score, were intentionally arranged at the right bottom corner. This group of genes together exhibited obvious signal at the tipping point (stage IIIA), and can be regarded as the dynamical network biomarker for distant metastasis of STAD. These top 1% genes with the most significant increase in local SSI scores were considered as the SSI-signaling genes which is a set of dynamical network biomarker and may highly relate to the catastrophic deterioration. Thus, we carried out functional analysis on these SSI-signaling genes.

Based on IPA analysis, the common SSI-signaling genes were highly related to functions annotation “Digestive organ tumor” (*P*-value = 3.0E-34), “Abdominal adenocarcinoma” (*P*-value = 7.1E-29), “Cancer of cells” (*P*-value = 2.2E-10), “Metastasis” (*P*-value = 2.0E-04), etc. Besides, from KEGG enrichment analysis, the SSI-signaling genes were enriched in cancer-related pathways including Pathways in cancer, AMPK signaling pathway, Ras signaling pathway. Some SSI-signaling genes have been found in literatures and identified to be associated with the process of cancer metastasis. For example, COL11A1 was reported as a remarkable biomarker for carcinoma progression and metastasis (Vázquez-Villa et al., 2015). BLNK was known as one of the downstream targets of Pax-5, which plays important role in metastasis (Crapoulet et al., 2011). HNRNPC, whose specific siRNA was reported to inactivate Akt pathway (Hwang et al., 2012) was also identified to control the metastatic potential of glioblastoma by regulating PDCD4 (Park et al., 2012). MMP1 proteolytically engage EGF-like ligands in an osteolytic signaling cascade for metastasis (Lu et al., 2009). LIN9 is a component of the metastasis-predicting Mammaprint gene signature in breast cancer (Van't Veer et al., 2002). The functional analysis showed that the SSI-signaling genes were highly related to metastasis or related biological functions, which also validated the sensitivity and effectiveness of the identified SSI-signaling genes. A list of common SSI-signaling genes for STAD was provided in Table S1.

### Identifying the Critical Transition for Influenza Infection

**Figure 5:**
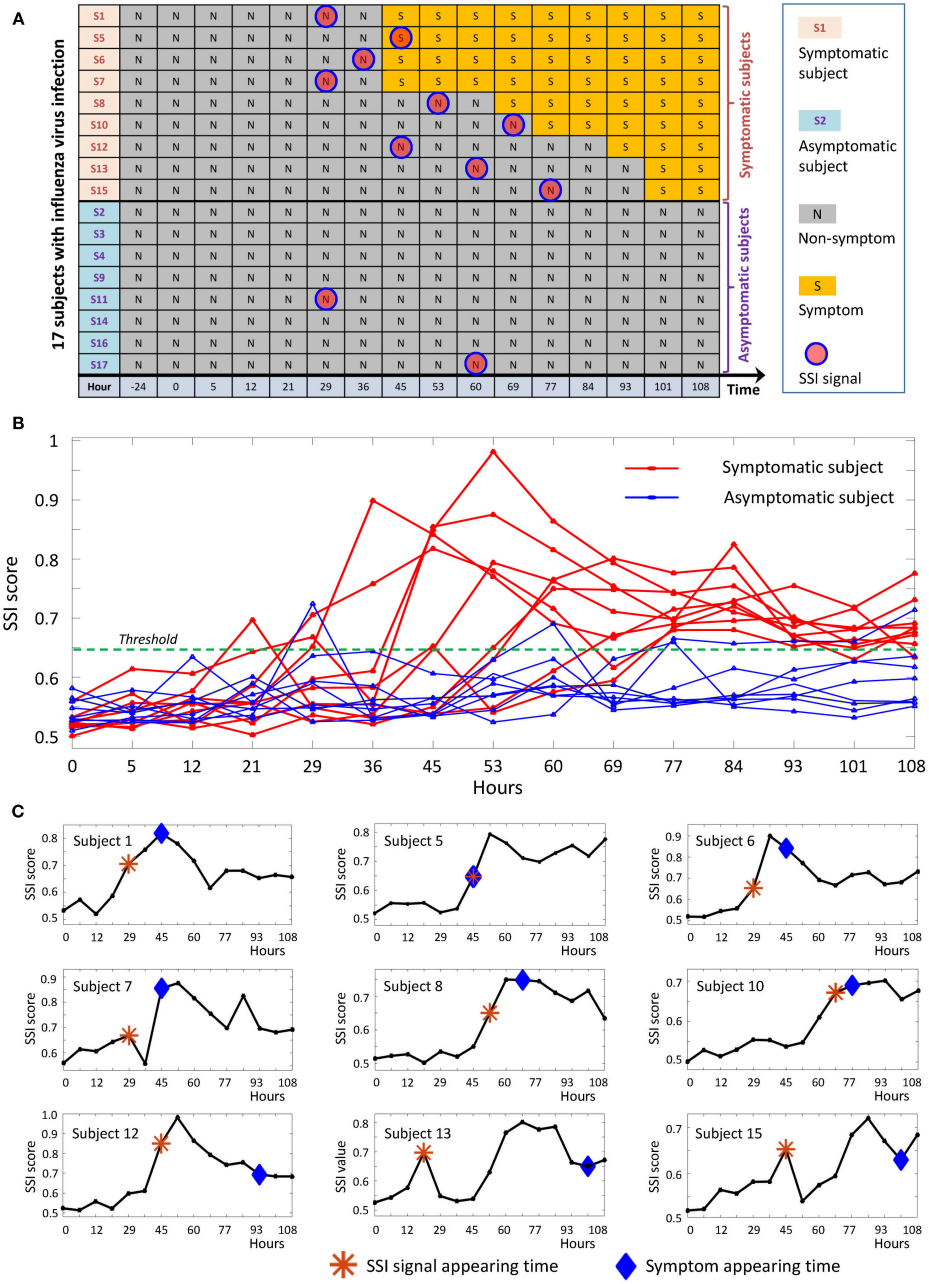
The application of SSI score in influenza infection dataset. **(A)** The overall information of the 17 subjects in the influenza-infection challenge. **(B)** Line chart of SSI score for all 17 subjects. The red curves are for symptomatic subjects, while the blue curves represent asymptomatic subjects. **(C)** The individual-specific SSI scores for 9 symptomatic subjects. For each SSI curve, the star symbol represents the time point when SSI-score signal arises, the diamond symbol represents the time point at which the initial flu symptoms appears.

We applied the proposed method to a time-course dataset of live influenza infection challenge (GSE30550), in which there were 17 subjects who received injection of influenza virus (H3N2/Wisconsin). Among the 17 subjects, nine (subjects 1, 5, 6, 7, 8, 10, 12, 13, and 15) were infected who showed clinic symptoms and the other eight (subjects 2, 3, 4, 9, 11, 14, 16, and 17) were always stay healthy who didn't show any clinic symptom during the whole period of infection challenge (Figure 5A). The gene expression profiles were derived in the whole peripheral blood drawn from all subjects at 16 time points, i.e., 24 h before injection, 0, 5, 12, 21, 29, 36, 45, 53, 60, 69, 77, 84, 93, 101, and 108 h after the injection. At each time point, there was only a single sample for each subject. By employing the proposed method, we obtained the individual-specific SSI score for each subject either in symptomatic or asymptomatic group.

**Figure 6:**
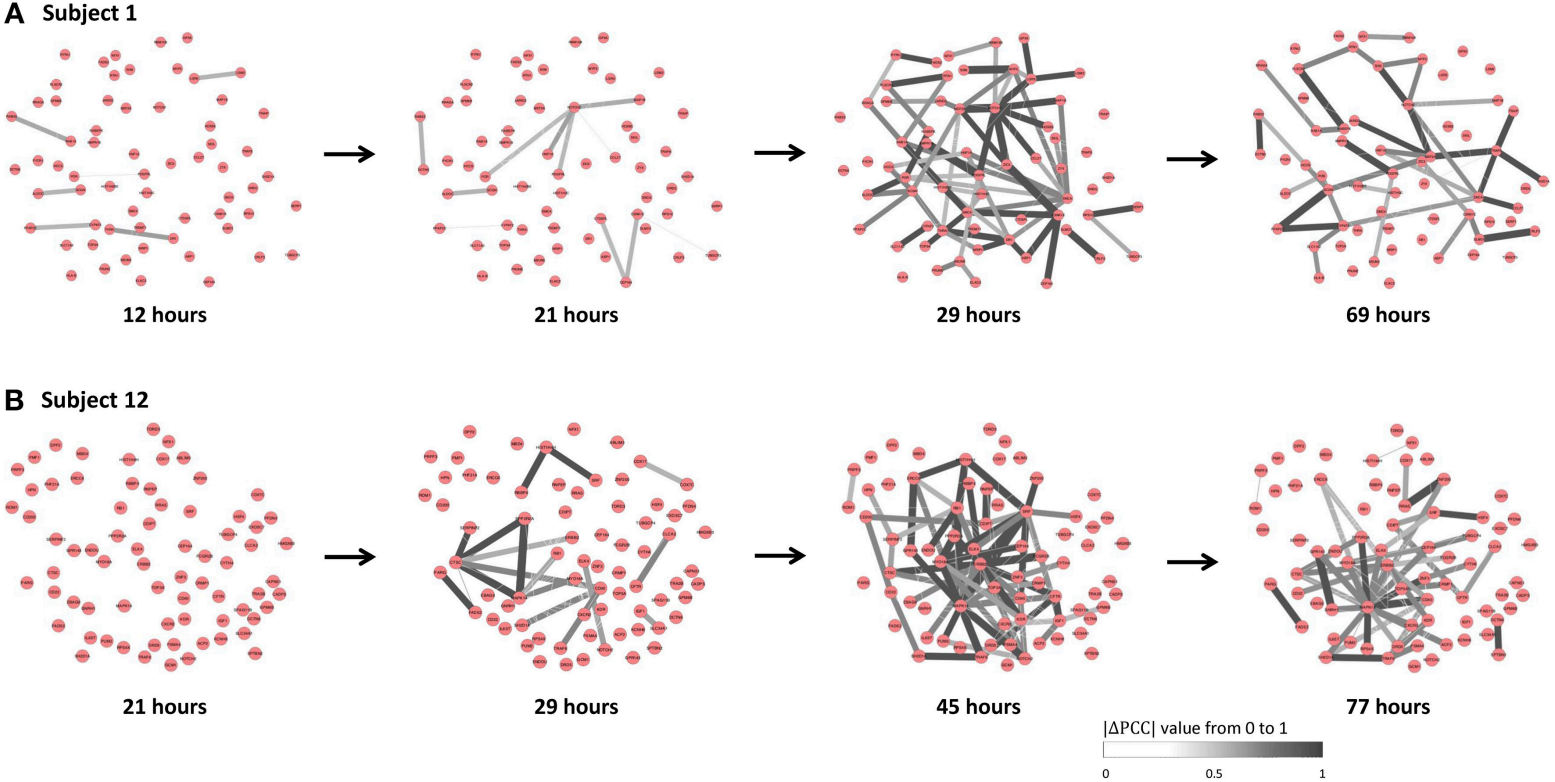
The dynamical evolution of subject-specific networks. To illustrate the dynamical evolution of the differential network, the individual-specific networks of two symptomatic subjects (subjects 1 and 12) were exhibited. **(A)** The individual-specific networks for subject 1. **(B)** The individual-specific networks for subject 12. Clearly, at the identified tipping point of each subject, there were considerably more differential edges than that at other time point.

The individual-specific SSI scores in Figure 5B demonstrated that there were obvious signals provided by SSI score for all symptomatic subjects (9 red curves), while there were few significant changes in the SSI scores for asymptomatic subjects (8 blue curves). The specific SSI scores for nine symptomatic subjects were shown in Figure 5C. Clearly, the SSI score indicated the pre-disease states (the state before the appearance of clinical symptom) for each symptomatic individual, with 100% accuracy. However, there was 25% false positive rate (Figure 5A). To demonstrate the evolution of individual-specific differential network, two sets of differential networks, respectively, for two symptomatic subjects, i.e., subject 1 and subject 12, were illustrated in Figure 6. Clearly, at the respective tipping point, there were many differential edges arising just before the emergence of clinic symptoms. At the tipping point of each symptomatic subject, the top 1% genes with the largest local SSI scores were regarded as a set of dynamical network biomarker, which were selected for further functional analysis.

Based on IPA analysis, the common SSI-signaling genes were highly related to functions annotation “Quantity of lymphocytes” (*P*-value = 2.23E-11), “Inflammation” (*P*-value = 2.47E-10), “Viral Infection” (*P*-value = 1.06E-09), “Homeostasis of leukocytes” (*P*-value = 1.14E-08). From KEGG enrichment analysis, the common SSI-signaling genes were enriched in Influenza A, and a variety of cellular pathways including PI3K-Akt signaling pathway, MAPK signaling pathway, NF-kappa B signaling pathway, etc. The functional analysis again validated the effectiveness of SSI-signaling genes. A list of common SSI-signaling genes for influenza infection was provided in Table S2.

## Discussion

Detecting the early-warning signal before a sudden deterioration into a severe disease state is crucial to patients all over the world. However, it is generally challenging to signal such critical transition through only a single case sample, since the lack of samples disables statistical indices and thus makes conventional methods fail. In this work, we proposed a computational method to identify the pre-disease state on the basis of a single sample. Specifically, given a number of reference samples which can be the normal samples of an individual (Figure 1A), the proposed method can distinguish the abnormal single sample by a differential-network-based HMM scheme. The proposed method has been validated by both the numerical simulation (Figure 3) and two real datasets (Figures 4, 5).

Comparing with the traditional methods which are mostly based on the differential expression of observed biomolecules, the proposed method aims at exploring the dynamic information of differential associations among biomolecules when a biological system is in the vicinity of a tipping point. This method thus possesses several obvious advantages. First, it works when only a single case sample is available, which benefits the analysis in personalized medicine. Second, it detects the pre-disease state rather than a disease state, which may help to achieve early diagnosis of some complex diseases. Third, it well-exhibits the critical properties at a network level which may provide new insights into catastrophic deterioration, such as the abnormally arising differential associations.

Although the proposed method is merely a step toward the identification of pre-disease state and the algorithm is expected to be improved in both sensitive and accurate ways, following the idea of personalized medicine, it provides a computational way and achieves individual-specific analysis and prediction by making use of only a single sample.

## Data Availability

Publicly available datasets were analyzed in this study. This data can be found here: https://www.ncbi.nlm.nih.gov/geo/query/acc.cgi?acc=GSE30550.

## Author Contributions

RL and PC conceived the project. PC supervised the project. JZ, XY, and YL performed computational and analysis. All authors wrote the manuscript and read and approved the final manuscript.

### Conflict of Interest Statement

The authors declare that the research was conducted in the absence of any commercial or financial relationships that could be construed as a potential conflict of interest.
