# Supplementary material for: Identifying Critical State of Complex Diseases by Single-Sample-Based Hidden Markov Model
Source: Front Genet. 2019 Apr 4;10:285. doi: 10.3389/fgene.2019.00285 (PMC6458292; doi:10.3389/fgene.2019.00285)
Supplement: Supplementary file 3 [file Data_Sheet_1.PDF]

Supplementary Information: Identifying critical state of complex diseases by single-sample-based hidden Markov model

Contents

We used a nine-gene network (see Fig. 3A in the main text) to conduct a numerical simulation for detecting the early-warning signals through SSI score. These types of gene regulatory networks are often used to study transcription, translation, diffusion, and translocation processes that affect gene regulatory activities (*1–5*). The following nine differential equations represent the gene regulation of nine genes in a network where gene regulation is represented in a Michaelis-Menten form, with the exception of the degradation rates, which are linearly proportional to the concentrations of the corresponding genes.

$$
\begin{cases}
\frac{\mathrm{d}z_1(t)}{\mathrm{d}t} = \frac{(8-4\,|p|)z_2(t)}{15\,(1+z_2(t))} - \frac{4+4\,|p|}{15}\,z_1(t) + \zeta_1(t), \\
\frac{\mathrm{d}z_2(t)}{\mathrm{d}t} = \frac{(4-2\,|p|)z_1(t)}{15\,(1+z_1(t))} - \frac{8+2\,|p|}{15}\,z_2(t) + \zeta_2(t), \\
\frac{\mathrm{d}z_3(t)}{\mathrm{d}t} = \frac{4\,|p|-10}{15} + \frac{5-2\,|p|}{15(1+z_1(t))} + \frac{5-2\,|p|}{15(1+z_2(t))} - z_3(t) + \zeta_3(t), \\
\frac{\mathrm{d}z_4(t)}{\mathrm{d}t} = \frac{4\,|p|-12}{15} + \frac{6-2\,|p|}{15(1+z_1(t))} + \frac{6-2\,|p|}{15(1+z_2(t))} - \frac{6}{5}z_4(t) + \zeta_4(t), \\
\frac{\mathrm{d}z_5(t)}{\mathrm{d}t} = \frac{4\,|p|-14}{15} + \frac{7-2\,|p|}{15(1+z_1(t))} + \frac{7-2\,|p|}{15(1+z_2(t))} - \frac{7}{5}\,z_5(t) + \zeta_5(t), \\
\frac{\mathrm{d}z_6(t)}{\mathrm{d}t} = -\frac{11}{15} + \frac{1}{15(1+z_1(t))} + \frac{1}{15(1+z_2(t))} + \frac{z_3(t)}{5(1+z_3(t))} + \frac{1}{5(1+z_5(t))} + \frac{1}{5(1+z_7(t))} \\
\qquad + \frac{1}{5(1+z_8(t))} - \frac{8}{5}\,z_6(t) + \zeta_6(t), \\
\frac{\mathrm{d}z_7(t)}{\mathrm{d}t} = \frac{z_8(t)}{10(1+z_8(t))} - \frac{19}{10}\,z_7(t) + \zeta_7(t), \\
\frac{\mathrm{d}z_8(t)}{\mathrm{d}t} = \frac{z_7(t)}{10(1+z_7(t))} - \frac{19}{10}\,z_8(t) + \zeta_8(t), \\
\frac{\mathrm{d}z_9(t)}{\mathrm{d}t} = -\frac{1}{10} + \frac{z_7(t)}{10(1+z_7(t))} + \frac{1}{10(1+z_8(t))} - \frac{11}{5}\,z_9(t) + \zeta_9(t),
\end{cases}
\tag{S1}
$$

where p is a scalar control parameter and $\zeta_i(t)\ \ (i = 1, 2, ..., 9)$ are Gaussian noises with zero means and covariances $\kappa_{ij} = \mathrm{Cov}(\zeta_i, \zeta_j)$. $z_i\ (i = 1, ..., 9)$ represent the concentrations of

mRNA-i. In Eq.(S1), the degradation rates of mRNAs are $(\frac{4+4|p|}{15}, \frac{8+2|p|}{15}, 1, \frac{6}{5}, \frac{7}{5}, \frac{8}{5}, \frac{19}{10}, \frac{19}{10}, \frac{11}{5})$. There is an equilibrium point $\bar{Z} = (\bar{z}_1, \bar{z}_2, ..., \bar{z}_9) = (0, 0, ..., 0)$. The differential equations Eq.(S1) can be transformed into the difference equations $Z(k+1) = f(Z(k), P)$ using the Euler scheme (*6*), *i.e.,*

$$\begin{cases} z_1(k+1) = z_1(k) + \left[\frac{(8-4\,|p|)z_2(k)}{15\,(1+z_2(k))} - \frac{4+4\,|p|}{15}\, z_1(k) + \zeta_1(k)\right]\Delta t, \\ z_2(k+1) = z_2(k) + \left[\frac{(4-2\,|p|)z_1(t)}{15\,(1+z_1(k))} - \frac{8+2\,|p|}{15}\, z_2(k) + \zeta_2(t)\right]\Delta t, \\ z_3(k+1) = z_3(k) + \left[\frac{4\,|p|-10}{15} + \frac{5-2\,|p|}{15(1+z_1(k))} + \frac{5-2\,|p|}{15(1+z_2(k))} - z_3(t) + \zeta_3(k)\right]\Delta t, \\ z_4(k+1) = z_4(k) + \left[\frac{4\,|p|-12}{15} + \frac{6-2\,|p|}{15(1+z_1(k))} + \frac{6-2\,|p|}{15(1+z_2(k))} - \frac{6}{5} z_4(k) + \zeta_4(k)\right]\Delta t, \\ z_5(k+1) = z_5(k) + \left[\frac{4\,|p|-14}{15} + \frac{7-2\,|p|}{15(1+z_1(k))} + \frac{7-2\,|p|}{15(1+z_2(k))} - \frac{7}{5}\, z_5(k) + \zeta_5(k)\right]\Delta t, \\ z_6(k+1) = z_6(k) + \left[-\frac{11}{15} + \frac{1}{15(1+z_1(k))} + \frac{1}{15(1+z_2(k))} + \frac{z_3(k)}{5(1+z_3(k))} + \frac{1}{5(1+z_5(k))} \right. \\ \qquad \left. + \frac{1}{5(1+z_7(k))} + \frac{1}{5(1+z_8(k))} - \frac{8}{5}\, z_6(k) + \zeta_6(k)\right]\Delta t, \\ z_7(k+1) = z_7(k) + \left[\frac{z_8(k)}{10(1+z_8(k))} - \frac{19}{10}\, z_7(k) + \zeta_7(k)\right]\Delta t, \\ z_8(k+1) = z_8(k) + \left[\frac{z_7(k)}{10(1+z_7(k))} - \frac{19}{10}\, z_8(k) + \zeta_8(k)\right]\Delta t, \\ z_9(k+1) = z_9(k) + \left[-\frac{1}{10} + \frac{z_7(t)}{10(1+z_7(t))} + \frac{1}{10(1+z_8(t))} - \frac{11}{5}\, z_9(t) + \zeta_9(t)\right]\Delta t, \end{cases} \tag{S2}$$

with a small time interval Δt. Note that $Z(k)$ is the vector of $Z(t)$ at the time instant $k\Delta t$.

We denote the Jacobian matrix of Eq.(S2) as $J = \left.\frac{\partial f(Z(k);p)}{\partial Z}\right|_{Z=\bar{Z}}$, where

$$J = e^{\Delta t \cdot A} \tag{S3}$$

with A is the coefficient matrix of the linearized system of Eq.(S1).

From Eq.(S3), we obtain nine distinct eigenvalues by taking $\Delta t = 1$, with the largest eigenvalue $0.67^{|p|} \to 1$ when $p \to 0$. Therefore, $p_c = 0$ is a critical value, at which there is a bifurcation of the system. Based on the simulated model Eq.(S2), we collected time-course data of the nine-node expressions and proceeded the calculation.

Besides, by using the simulated dataset generated from Eq. (S1), we have the following discussion.

As shown in Fig. S1, we have analyzed the signals under different reference condition. It is seen from Figs. S1A and C that when the system is perturbed by only a small noise, the signal

yielding from a small number of reference samples is as good as that from many reference samples. However, when the system is perturbed by a large noise, the signal is smoother and more distinguishable when there are a lot of reference samples. Therefore, a sufficient set of reference samples is considered as one of the guarantees of a robust signal, to against noise perturbation or data error.

As shown in Fig. S2, we have analyzed the signals when threshold d varies. It is seen from Fig. S2 that although the strength of signal may change, the SSI-score accurately indicate the tipping point with similar tendency. Therefore, different settings of threshold d may affect the strength of the signal, but do not affect the evolution tendency (e.g., abrupt increase when approaching the tipping point) of the signal curve.

As shown in Fig. S3, we have also provided an SSI score curve, with a new parameter value $p = 0$ instead of the value $p = -0.001$. It exhibits that the tendency of the new SSI score curve is similar to that in Fig. 3B in the main text, which demonstrate that the SSI score still works when a sampling point is exactly at the critical point.

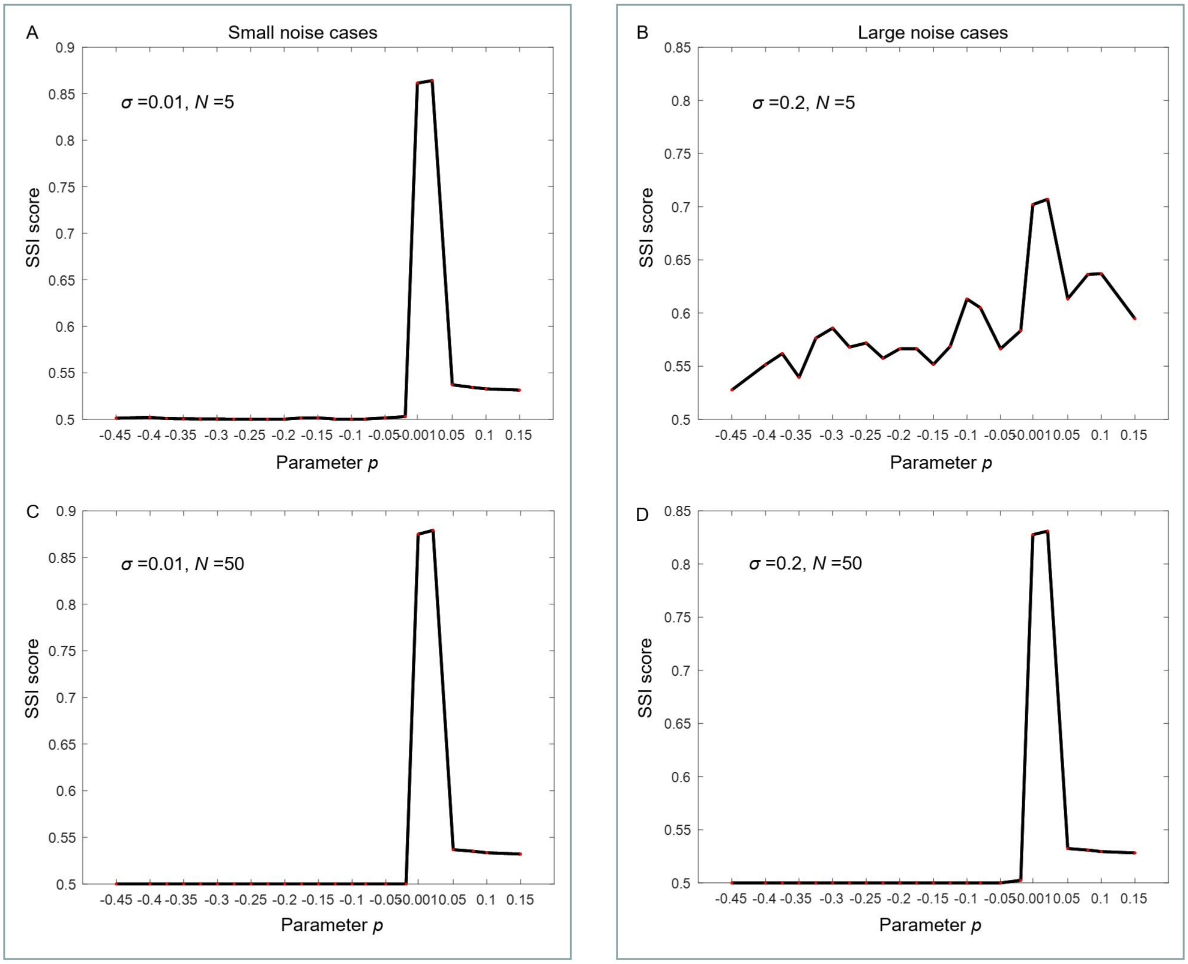

Figure S1: | **The critical signals under different reference sample sizes** Based on the numerical simulation dataset generated from Eq. (S1) , whose structure is shown in Fig.3A in the revised manuscript, and corresponding stochastic equation set and calculation details are provided in the revised Supplementary Information, we analyzed the signals (i.e., the so-called SSI score) to the critical transition of the network system with different reference samples sizes. (A) The SSI-score curve when noise strength $\sigma = 0.01$ (the small noise case) and the number of reference samples $N = 5$. (B) The SSI-score curve when noise strength $\sigma = 0.2$ (the large noise case) and the number of reference samples $N = 5$. (C) The SSI-score curve when noise strength $\sigma = 0.01$ (the small noise case) and the number of reference samples $N = 50$. (D) The SSI-score curve when noise strength $\sigma = 0.2$ (the large noise case) and the number of reference samples $N = 50$. It is seen that when the system is perturbed by only a small noise, the signals are similar when the reference sample size varies. However, when the system is perturbed by a large noise, the more reference samples there are, the smoother and more accurate the signal is.

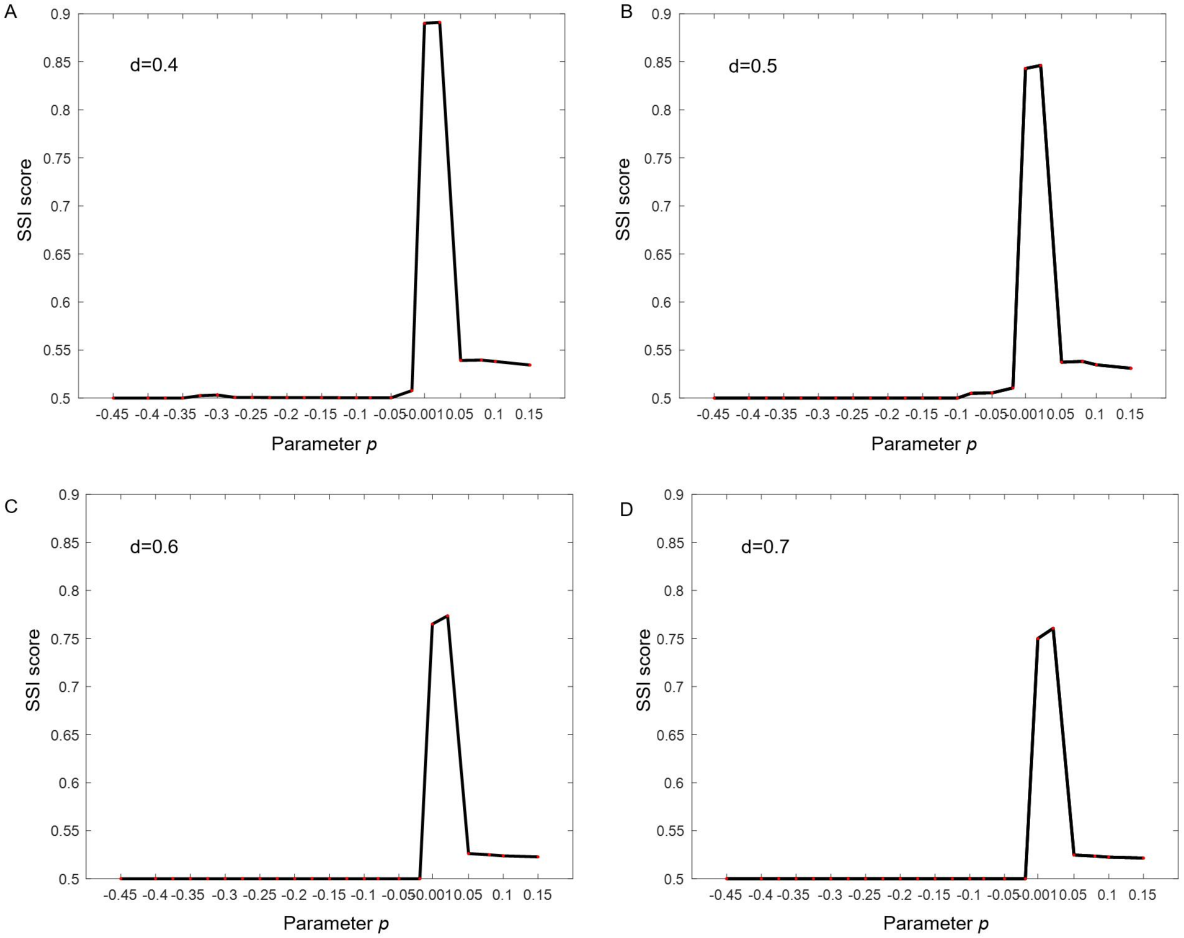

Figure S2: | **The critical signals under different threshold settings** Also based on the numerical simulation dataset, we analyzed the signals to the critical transition with different values of threshold d. (A) The signal curve for $d = 0.4$. (B) The signal curve for $d = 0.5$. (C) The signal curve for $d = 0.6$. (D) The signal curve for $d = 0.7$. It is seen that when the value of threshold d varies, the SSI-score curves accurately indicate the tipping point with similar tendency, although the strength of signals may change.

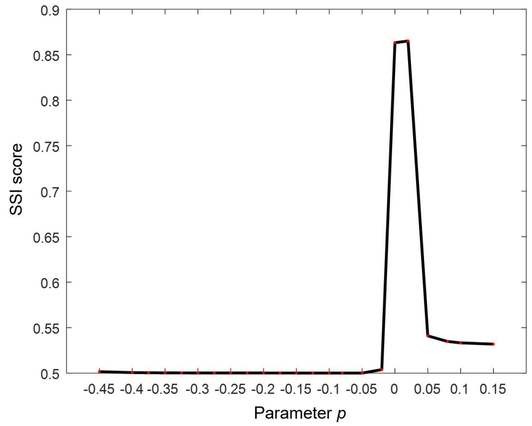

Figure S3: | **The single-sample inconsistency (SSI) score curve with a parameter value $p = 0$.** In the x-axis, there is a new parameter value $p = 0$ instead of the original $p = -0.001$. It is seen that the tendency of the new SSI score curve is similar to the original one, which demonstrate that the SSI score still works when a sampling point is exactly at the critical point.

References

1. Chen, L., Wang, R., Li, C. & Aihara, K. *Modeling Biomolecular Networks in Cells: Structures and Dynamics*, (Springer, New York, 2010).

2. Chen, L., Wang, R. & Zhang, X. *Biomolecular Networks: Methods and Applications in Systems Biology*, (John Wiley & Sons, Hoboken, New Jersey, 2009).

3. Becskei, A. & Serrano, L. Engineering stability in gene networks by autoregulation, *Nature* **405**, 590–593(2000).

4. Chen, L. & Aihara, K. Stability of genetic regulatory networks with time delay, *IEEE Trans. Circuits Syst. I* **49**, 602–608(2002).

5. Li, C., Chen, L. & Aihara, K. Stability of genetic networks with SUM regulatory logic: Lur'e system and LMI approach, *IEEE Trans. Circuits Syst. I* **53**, 2451–2458(2006).

6. Kloeden, P. & Platen, E. *Numerical Solution of Stochastic Differential Equations*, (Springer, 1999).
